# Voice-Based Pain Level Classification for Sensor-Assisted Intelligent Care †

**DOI:** 10.3390/s26030892

**Published:** 2026-01-29

**Authors:** Andrew Y. Lu, Wei Lu

**Affiliations:** 1 Oyster River High School, Durham, NH 03824, USA; 27lua@orcsd.org; 2 Department of Computer Science, Keene State College, Keene, NH 03431, USA

**Keywords:** pain level classification, zero-effort technology, healthcare AI, acoustic sensor, Convolutional Neural Network (CNN), MFCC, spectrogram

## Abstract

Various sensors are increasingly being adopted to support intelligent healthcare systems, which address the growing problem of staff shortages in assisted-living communities. In this context, detecting and assessing pain remain critical yet challenging tasks in both clinical and non-clinical settings. Traditional approaches such as self-reporting, physiological signal monitoring, and facial expression analysis often face limitations related to accessibility, equipment costs, and the need for professional support. To overcome these challenges in this work, we investigate a sensor-assisted system for pain detection and propose a lightweight framework that enables real-time classification of pain levels using acoustic sensors. Our system exploits the spectral features of voice signals that strongly correlate with pain to train Convolutional Neural Network (CNN) models. Our system has been validated through simulations in Jupiter Notebook and a Raspberry Pi-based hardware prototype. The experimental results demonstrate that the proposed three-level pain classification approach obtains an average accuracy of 72.74%, outperforming existing methods with the same pain-level granularity by 18.94–26.74% and achieving performance comparable to that of binary pain detection methods. Our hardware prototype, built from commercial off-the-shelf components for under 100 USD, achieves real-time processing speeds ranging from approximately 6 to 22 s. In addition to CNN models, our experiments demonstrate that other machine learning algorithms, such as Artificial Neural Networks, XGBoost, Random Forests, and Decision Trees, also prove to be applicable within our pain level classification framework.

## 1. Introduction

To address the pressing staff shortages in assisted-living communities, a variety of sensors are being integrated into intelligent care systems to support independent living, health monitoring, and early intervention [1,2]. Among the many different health monitoring methods, pain detection and early management have a significant impact on treatment outcomes. Accurately assessing a patient’s pain is essential for effective treatment planning, medication dosing, and ensuring patient comfort.

**Figure 1:**
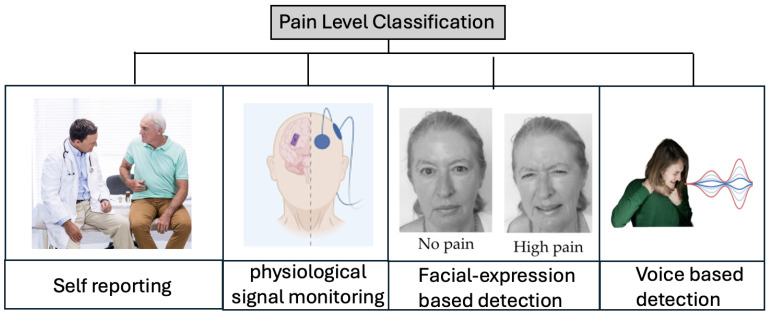
Categories of existing pain level classification methods.

The classification of pain levels remains a fundamental challenge in both clinical and non-clinical healthcare settings. Traditionally, pain is reported through self-assessment questionnaires or verbal reports. However, self-reporting methods are subjective and infeasible for nonverbal patients, leading to under-reporting or exaggeration [3]. To address this limitation, researchers have explored several pain level classification techniques, which can be broadly categorized into four types, as shown in Figure 1: self-reporting, physiological signal monitoring, facial expression analysis, and voice-based detection.

Physiological methods rely on biosignals, such as heart rate variability, electroencephalography, electromyography, galvanic skin response, and blood pressure readings, to infer pain states [4]. Although effective, such methods often require specialized equipment and controlled environments, making them impractical for daily use or low-resource settings. Facial-expression-based approaches utilize computer vision and machine learning algorithms to infer pain from facial muscle movements [5,6]. However, their accuracy is highly dependent on lighting conditions, camera angle, and the absence of occlusions or background noise, which limit their robustness in real-world deployment [7].

Recently, voice-based pain level classification has emerged as a promising alternative due to its non-invasive nature and low cost [8]. Voice signals captured by simple microphones are sufficient for audio analysis in low-end edge devices. The literature indicates that vocal signals—both verbal and nonverbal—carry biometric and emotional cues that correlate with physical discomfort [9]. However, existing audio-based systems often rely on the analysis of static and pre-recorded datasets and lack real-time processing capabilities [10], making them unsuitable for daily use. The best performance achieved in [11,12,13] was limited to binary pain detection.

To address these limitations, we propose a lightweight, portable, and real-time framework for pain level classification. Our method can analyze both verbal and nonverbal audio inputs to classify pain levels as low, moderate, or high. This article is a revised and expanded version of our conference paper [14]. In this work, we provide more details in our analysis, method description, and experimental results. More specifically, this work makes the following key contributions:

- Rather than requiring the process of loading pre-recorded audio clips with human annotations, we propose an automated pain-level classification framework that leverages acoustic sensor data and pretrained Convolutional Neural Network (CNN) models from publicly available pain datasets to classify three pain levels.
- Our framework enables a comprehensive investigation of the most relevant spectral features in verbal and nonverbal audio signals to improve the accuracy of the CNN model for pain level classification. Our framework validates the acoustic distinguishability of different pain levels and the interpretability of our CNN models. Moreover, our study shows that different pain levels are strongly correlated with spectral features, including pitch, formants, and energy distribution in high-frequency bands.
- We implemented the proposed method in a Python-based (version 3.12) app for a local computer or a web-based assessment portal. We also developed a low-cost hardware prototype with commercial off-the-shelf devices, achieving low latency (<22 s). Our prototype demonstrates its feasibility for edge healthcare applications.
- The proposed pain-level classification method is evaluated with a verbal audio dataset (TAME) and a nonverbal audio dataset (VIVAE). The average accuracy, false negative rate, the impact of imbalanced samples on accuracy, and inference time are thoroughly compared with existing methods.
- Besides CNN models, other machine learning algorithms such as Artificial Neural Networks (ANN), XGBoost, Random Forest (RF), and Decision Tree (DT) have also proven applicable within our pain level classification framework.

The rest of the paper is organized as follows: Section 2 analyzes the correlation between different pain levels and spectral features of audio data. Section 3 introduces the proposed methodology and system architecture. Section 4 presents experimental results, including performance under biased datasets and model comparisons. Section 5 introduces a possible deployment in real-world scenarios. Section 6 concludes the paper and outlines future directions.

## 2. Correlation Analysis of Pain Level and Spectral Features

To study the characteristics of acoustic sensor data, spectral analysis is a common tool since it describes how sound energy is distributed across frequency at a given moment. In this work, we exploit spectral features to correlate the pain levels and the verbal/nonverbal audio data.

Different pain levels affect how the body produces the voice. For instance, pain often leads to tightening of the throat muscles, diaphragm, and chest wall, and pain can also cause strain or constriction in the larynx. Due to the pain, the air pressure from the lungs also increases. As a result, the vocal folds may vibrate more irregularly or more forcefully. More pain could also lead to a tighter vocal fold, which produces a voice with a higher pitch (i.e., louder sound).

From an acoustic perspective, these physiological changes are reflected in several commonly used spectral features. Pitch (fundamental frequency, F0) captures the vibration rate of the vocal folds and is closely related to perceived voice height. Formant frequencies (F1, F2, etc.) characterize the resonant properties of the vocal tract and are sensitive to changes in articulation, tongue position, and jaw tension. Spectral energy distribution and spectral centroid describe how acoustic energy shifts across frequency bands and are often associated with vocal effort and tension. Together, these features provide a quantitative representation of how pain-induced physiological responses manifest in voice signals.

**Figure 2:**
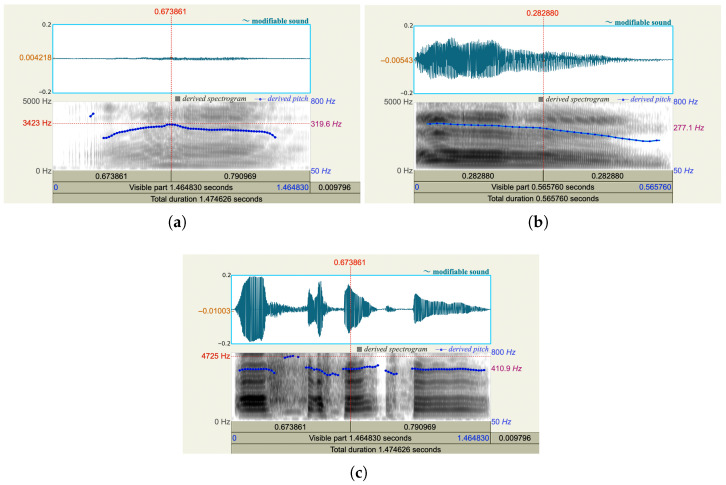
Comparison of the spectrogram for the voice from a subject experiencing (**a**) low level pain, (**b**) moderate level pain, and (**c**) high level pain.

**Figure 3:**
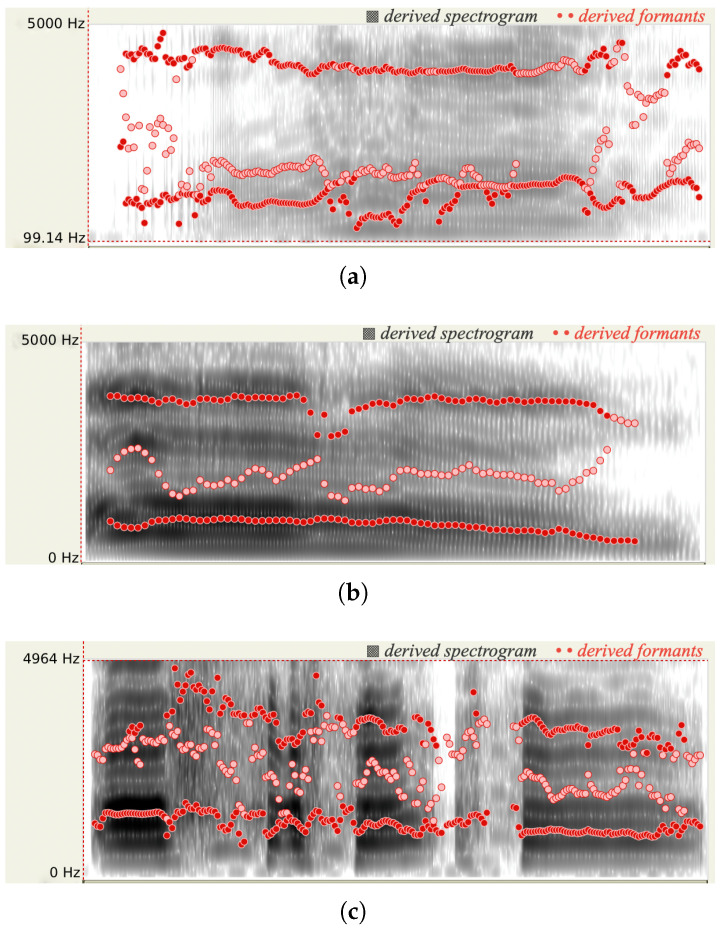
Formants shift due to (**a**) low, (**b**) moderate, and (**c**) high level pain. The red dot lines represent three formants varying with time. Note that all spectrograms were obtained from Praat [15].

We used the software Praat [15] to analyze three vocal recordings from the same person who was experiencing low, moderate, and high pain, respectively. As shown in Figure 2, when the person has more pain, more energy occurs in the high-frequency range, the spectrum is broader, and a higher pitch is derived. When a person experiences pain, his/her may clench jaw and retract tongue. Consequently, he/she could lose control of articulation. As shown in Figure 3, the formant frequencies for the audios with three pain levels shift.

**Figure 4:**
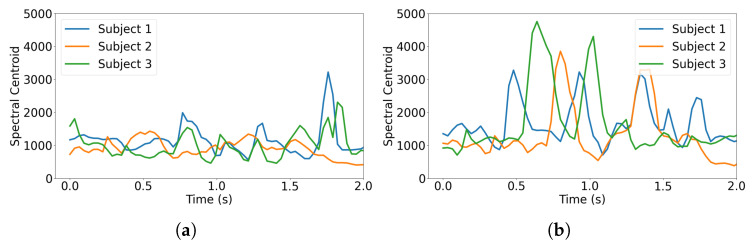
Spectral centroid for the voice from three subjects experiencing (**a**) low-level pain and (**b**) high-level pain.

Existing literature [9,16] also confirms that some spectral features for an audio signal have high correlation with the subject’s pain level. Our analysis framework enables a detailed comparison of voice signal characteristics across time and frequency domains. Our preliminary study based on the TAME dataset [17] indicates that different pain levels result in varying intensities in the spectrogram (shown in Figure 3) and spectral centroid frequency (shown in Figure 4). In the following section, we incorporate the highly correlated spectral features into our CNN training models.

## 3. Proposed Framework for Three-Level Pain Classification Using Spectral Cues in Verbal and Nonverbal Voice

### 3.1. Overview of Our Framework

**Figure 5:**
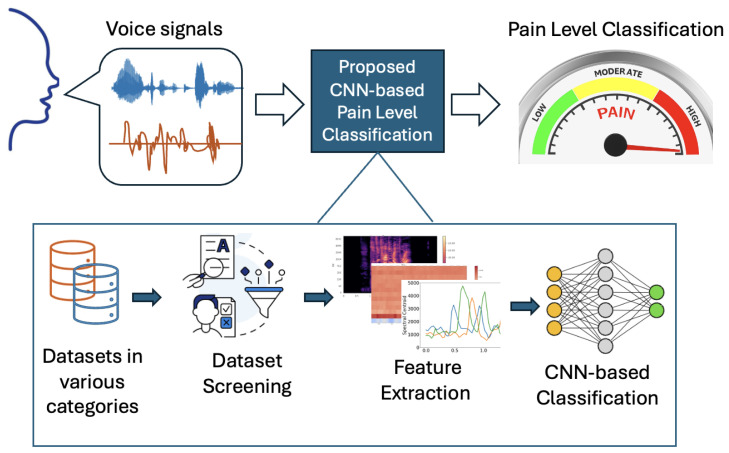
Overview of proposed voice-based pain level classification framework.

To support pain management in non-clinical, resource-constrained settings (e.g., home care or bedside monitoring in daily use), we propose a low-cost framework that classifies pain levels by analyzing voice signals in real time. Our framework enables automating the feature extraction from a real-time audio stream and classifying the corresponding pain level by inferring from our pre-trained CNN models. To examine the impact of unbalanced clinical pain datasets (i.e., bias) on prediction accuracy, our framework also facilitates training CNN models with various datasets, including verbal and non-verbal audio files labeled with gender and age. As shown in Figure 5, our framework consists of the following core stages: audio acquisition, preprocessing and feature extraction, CNN-based classification, and result visualization.

### 3.2. Software Design

**Figure 6:**
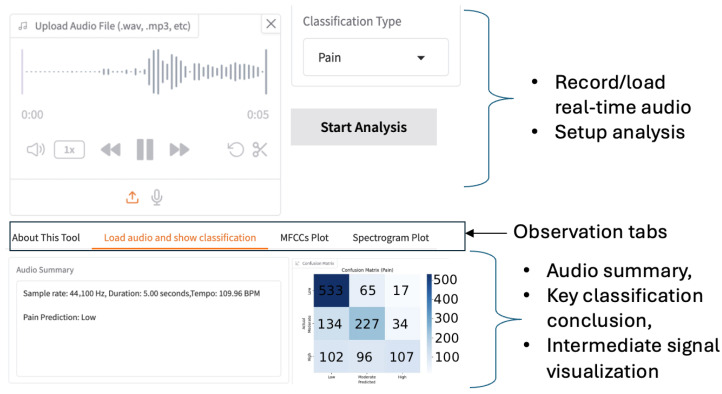
Interface of our training and testing tool for pain level classification.

Our software implementation is feasible on a local computer or via a web-based portal, allowing users to record audio in real time or upload pre-recorded audio files. Figure 6 illustrates the graphic interface of our app. To trade off the memory space, processing speed, and classification accuracy, the duration and audio sample rate are reconfigurable. The default duration is five seconds, and the typical sample rate is 44.1 kHz. The voice-capture interface supports audio replay and trimming before subsequent feature extraction. As a result, users have fine-grained control over real-time audio during inference. The results of the analysis performed by our framework are displayed in a multi-tab panel, as shown in Figure 6. Based on the classification objective, the control panel guides users in selecting the most appropriate trained CNN model. For batch testing with multiple audio files, the system visualizes performance using a confusion matrix. For a single audio file, the tool outputs the predicted pain level directly. By visualizing spectral features, we enhance interpretability and speed up the optimization of CNN models.

On the backend, massive datasets (e.g., TAME [17] and VIVAE [18]) have been used to train our CNN models. Our system automatically generates spectrograms and extracts spectral features, including Mel-Frequency Cepstral Coefficients (MFCCs), pitch, formants, low-band energy ratio, spectral centroid, and zero-crossing rate, as possible inputs for neural network training. We employ librosa 1.22, tensorflow 2.20, and sklearn 1.7 packages to extract features and build neural network models. More details are available in Algorithm 1.

CNN was selected as the primary model for this work based on both empirical results and task-specific considerations. First, the input to our framework is a two-dimensional time–frequency representation (mel spectrogram) transformed from raw audio signals, which naturally aligns with the convolutional architecture of CNNs for spatial feature extraction. Second, CNN demonstrated more stable performance across pain levels compared to classical machine learning models, particularly when modeling spectral patterns. Finally, the adopted CNN architecture was intentionally designed to be lightweight, achieving a favorable balance among classification accuracy, robustness, and computational complexity, which is critical for real-world deployment on resource-constrained devices. The CNN model consists of the following layers:

- **Input layer** accepts MFCC/spectrogram tensor (e.g., 128×128×1).
- **Convolutional blocks** include two to three convolutional layers with ReLU activation and max-pooling.
- **Flatten + Dense layer** is reduced to a feature vector for classification.
- **Output layer** is a softmax layer that produces the probability distributions over the three pain classes: low, moderate, and high.

**Algorithm 1.** Pseudocode for the CNN Model Training in Audio-Based Three-Level Pain Classification**Require:** 
Audio datasets AD, CNN model configuration parameters Mconfig, List of spectral features strongly correlated with pain *L***Ensure:** 
pretrained CNN model *M*  1:import librosa  2:import tensorflow  3:import sklearn packages  4:load AD  5:Reshape AD into segments for the given CNN with Mconfig  6:Extract Melspectrum  7:**for** each audio segment **do**  8:      Compute spectral features in *L*  9:      Transform feature representation10:      Train and refine CNN model *M* with selected spectral features11:**end for**12:**return** *M*

The depth of the CNN and the associated training parameters were selected based on preliminary empirical evaluations to balance classification performance and computational efficiency, consistent with the lightweight deployment goal of this framework. After training the CNN, we use the algorithm presented in Algorithm 2 to predict the pain level from the captured audio. As we separately train the CNN with the verbal audio dataset (TAME) and the nonverbal audio dataset (VIVAE), users need to select the pretrained CNN model accordingly for their input.
**Algorithm 2.** Pseudocode for Inference in Audio-Based Three-Level Pain Classification**Require:** 
Real-time audio input RA, pretrained neural network model *M***Ensure:** 
Predicted pain level P∈{Low,Moderate,High}  1:Acquire raw audio signal using acoustic sensors  2:**if** len(RA) less than the minium threshold **then**  3:      Pad audio stream with 0  4:**else**  5:      Segment audio signal into short-time frames  6:**end if**  7:**for** each audio segment **do**  8:      Compute spectral features SF  9:      Transform spectral features $SF^{\prime }$10:      Feed $SF^{\prime }$ into pretrained neural network model *M*11:      Obtain pain-level probabilities12:**end for**13:Aggregate segment-level predictions14:Determine final pain level *P*15:**return** *P*

### 3.3. Hardware Prototype

**Figure 7:**
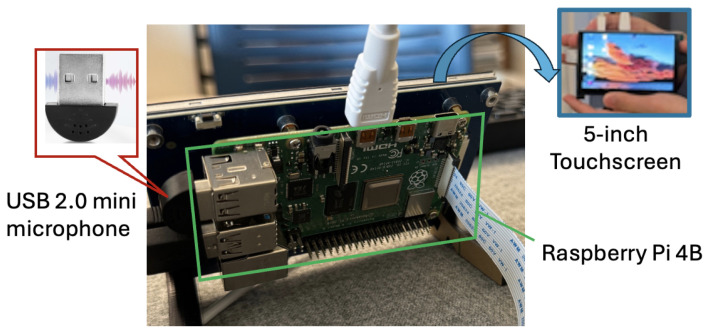
Our hardware prototype.

The CNN training model and inference engine have been successfully deployed on a hardware prototype consisting of a USB 2.0 mini microphone for audio acquisition, a Raspberry Pi 4B for real-time analysis and classification, and a 5-inch MIPI DSI touchscreen for interactive system control and result visualization. The microphone (i.e., acoustic sensor) weighs only 3 g and has dimensions of 22.0 mm × 19.0 mm × 7.0 mm. The noise ratio of the sensor is greater than 67 dB. Its frequency response range is between 100 Hz and 16 kHz. The Raspberry Pi was programmed with Python 3.11.9, TensorFlow 2.20, and sklearn libraries. Figure 7 shows the overview of our prototype.

## 4. Experimental Results

### 4.1. Experimental Setup

We evaluate the proposed framework by testing its accuracy in differentiating three pain levels using a verbal speech dataset, TAME [17], which contains 624 high-pain samples, 2213 moderate-pain samples, and 4202 low-pain samples, and a nonverbal audio dataset VIVAE [18], which has 1085 audio files in total. The datasets we adopted in this work have an explicit label for how the subjects rate their pain level from 0 to 10. We categorized the pain level 0–4 as low, 5–7 as moderate, and 8–10 as high. The training datasets include samples from subjects aged 18 to 33 and represent both genders. The configuration for the CNN architecture is the same as described in Section 3.2.

### 4.2. Classification Accuracy

#### 4.2.1. Average Accuracy of Pain Level Classification

**Figure 8:**
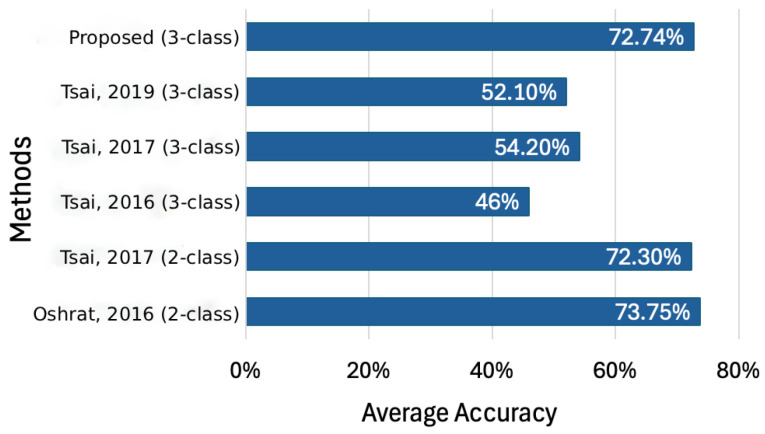
Comparison of the accuracy achieved by different pain level classification methods, including the proposed three-class approach and prior two-class and three-class methods reported by [11,12,13,19].

The following experiments randomly chose samples from the TAME pain dataset and were performed in Visual Studio Code 1.97. We evaluate the performance of our approach against several existing methods introduced in [11,12,13,19]. While refs. [11,13] report accuracy rates exceeding 70%, they are restricted to binary classification tasks. By contrast, our model, which distinguishes between three pain levels, achieves an average accuracy of 72.74%, as illustrated in Figure 8. This result is on par with the two-class systems proposed in [11,13], and surpasses the performance in [12] and [19] by 26.7% and 20.6%, respectively.

#### 4.2.2. Comparison of False Negative Rates for Audio- and Facial Expression-Based Pain Detection

**Figure 9:**
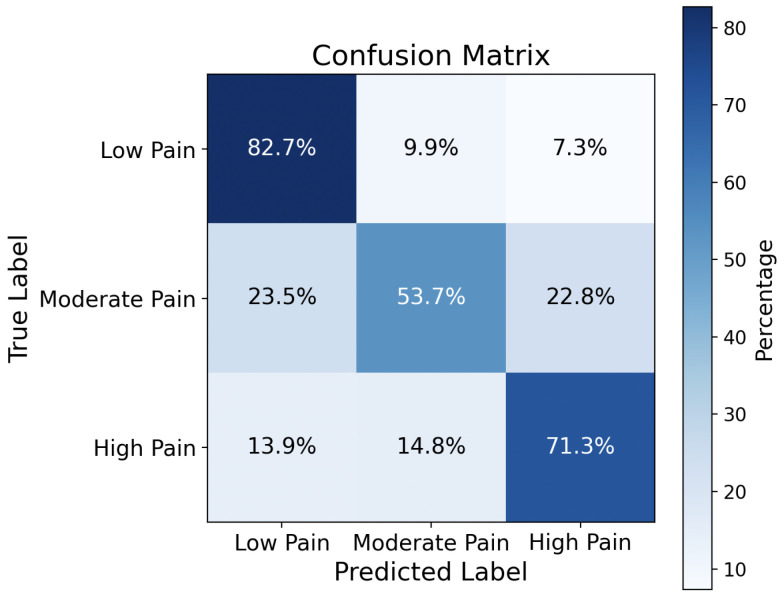
Confusion matrix achieved by the CNN trained by the proposed method with the verbal audio dataset TAME.

False Negative Rate (FNR) is an important metric to examine if the detection method will miss important true cases. In this section, we compare the proposed audio-based pain level classification with a facial-expression-based pain detection method [20]. The evaluation of the facial-expression-based approach was performed using images from the MIntPain [21] database. As reported in [20], the false negative rate of the facial-expression-based method ranges from 0% to 36%, depending on the specific action unit used as a cue. Figure 9 presents the confusion matrix obtained from the CNN model trained using the proposed method. From this confusion matrix, the false negative rates for low, moderate, and high pain are 17.3%, 46.3%, and 28.7%, respectively. The false negative rate comparison is summarized in Table 1. The FNR for low and high pain levels is comparable with facial expression-based pain detection.

#### 4.2.3. Comparison of Classification Accuracy for Verbal and Nonverbal Audios

**Figure 10:**
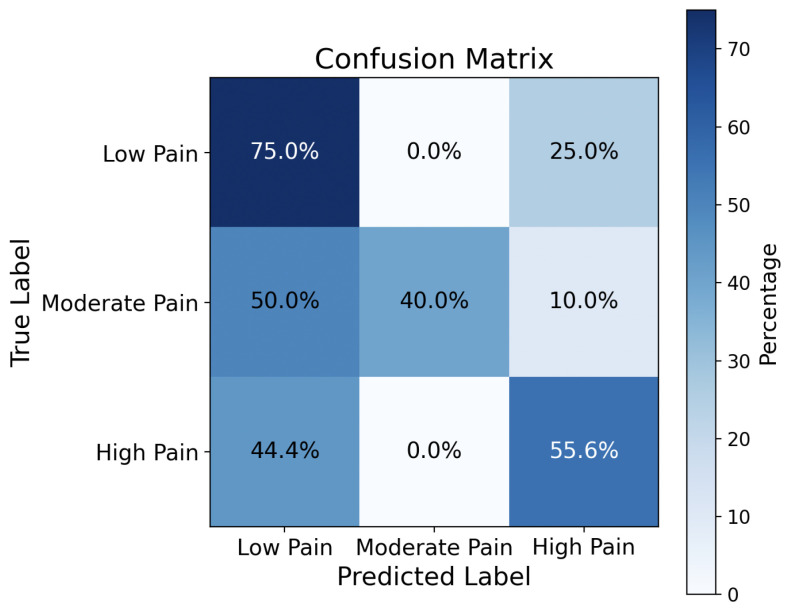
Confusion matrix achieved by the CNN trained by the proposed method with the nonverbal audio dataset VIVAE.

The CNN model trained by the verbal dataset (TAME) allows us to achieve an average pain classification accuracy of 72.74%. In contrast, the CNN model trained by the nonverbal dataset (VIVAE) achieves only 55.56%, which is 1.31× less than the verbal speech. Figure 10 shows the confusion matrix. This is because nonverbal sounds have only signal roughness as intensity, resulting in less intensity in log-Mel spectrogram and zero-crossing rate than the verbal speech. Another important critical reason is VIVAE is a smaller dataset than TAME.

### 4.3. Impact of the Number of Training Samples on Classification Accuracy

**Figure 11:**
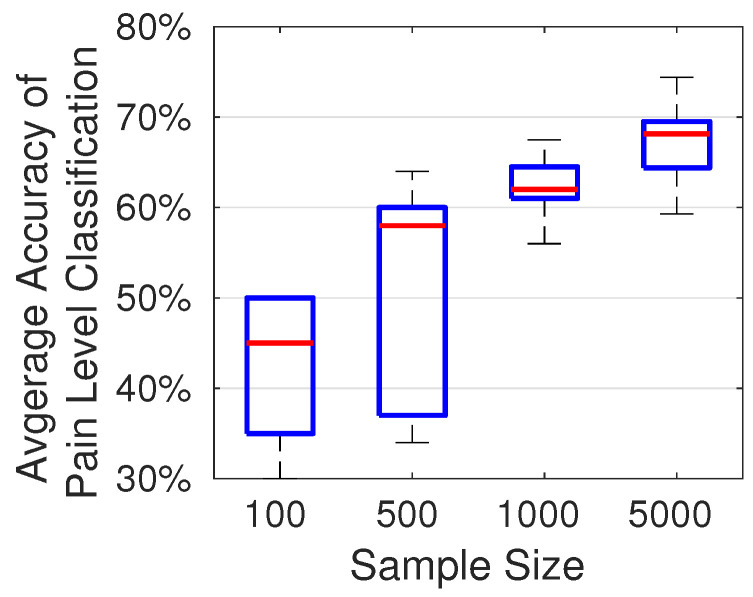
Accuracy of pain level classification achieved by the CNN models trained on different numbers of samples.

The accuracy of pain level classification depends on the number of samples used in training the CNN model. To quantitatively assess the impact of the number of training samples on accuracy, we randomly selected subsets of 100, 500, 1000, and 5000 audio samples from the TAME pain dataset. For each subset, the proportions of low, moderate, and high pain samples were maintained in accordance with their distribution in the full TAME pain dataset. As shown in Figure 11, more training samples generally produce a more robust CNN model and an improved classification performance. When trained with over 7000 samples, our model achieves an accuracy of up to 74%. The variation in accuracy is due to the random distribution of the selected audio samples. The results shown in Figure 11 are based on 10 trials for each sample size.

**Figure 12:**
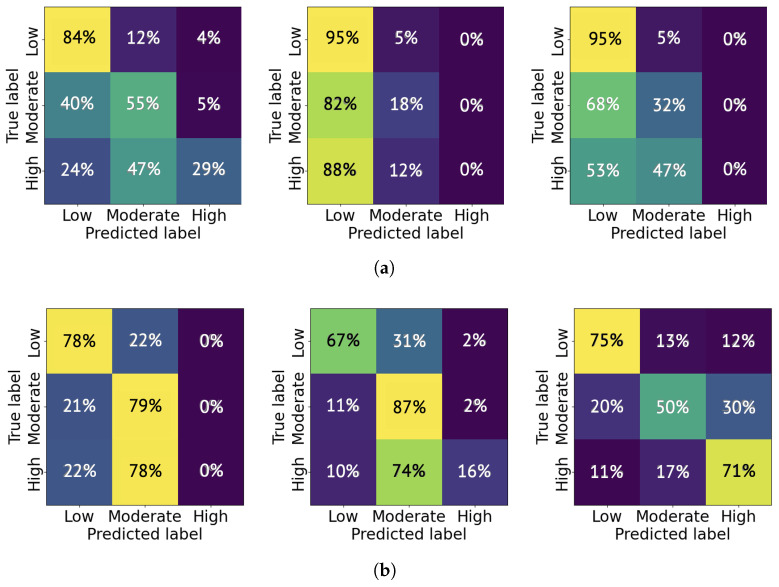
Three trials’ confusion matrix for pain level classification using a dataset with (**a**) 1000 samples and (**b**) 5000 samples randomly selected from the TAME pain dataset.

The average classification accuracy is a product of the classification accuracy for a specific pain level and the percentage of that pain level’s samples distributed in the total testing cases. As an example, we present the confusion matrices from six trials: three trained on 1000 samples and three trained on 5000 samples. As shown in Figure 12a, the CNN model tends to classify the pain level as low more significantly than moderate and high, and the high pain level is rarely labeled. This indicates a conservative tendency in the model’s pain level estimation. The likely cause of this behavior is the class imbalance in the selected 1000 samples from the TAME pain dataset, where low-pain samples constitute the majority. When using 5000 samples, the trained CNN model achieved an improved accuracy in classifying high-level pain. For instance, as shown in Figure 12b, the rightmost trial demonstrates a high-to-high classification accuracy of 71%. The experiments were repeated across 10 independent trials, and the average probability of correctly classifying each pain level is reported in Table 2. As shown, the classification accuracy for moderate and high pain levels generally improves with an increased number of training samples.

### 4.4. Impact of Biased Samples on Classification Accuracy

As observed from the previous subsection, a biased sample distribution in the training data can lead to significant inaccuracies in real-world applications. When certain classes are overrepresented—such as low pain levels in pain classification—the model tends to favor these dominant classes, resulting in reduced sensitivity and accuracy for underrepresented categories, such as moderate and high pain levels. Motivated by this observation, we employ distinct methods to quantitatively assess the impact of label-level and feature-level bias techniques on the accuracy of pain classification models.

**Figure 13:**
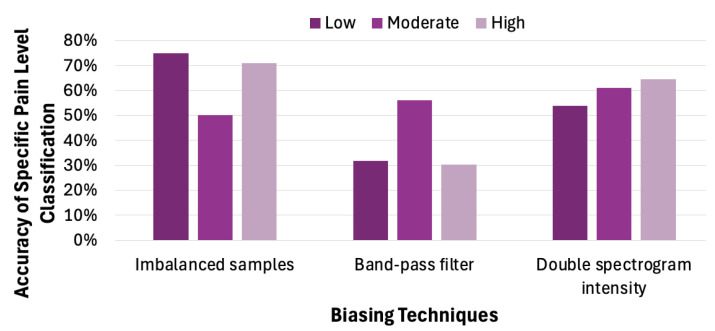
Impact of biasing methods on the classification accuracy for a specific pain level.

Label imbalance refers to an uneven distribution of class labels in a training dataset. In our TAME pain dataset, the labels are imbalanced (the low-pain class is the majority). To analyze how label imbalance influences model behavior and classification accuracy, we randomly chose 1000 samples from the TAME dataset, following the label distribution shown in Table 3. We used those samples to train a CNN model and created a balanced sample set for accuracy testing. When high pain cases dominate the training samples, the model overwhelmingly predicts high pain, regardless of the true label. The low and moderate pain levels are almost entirely incorrectly classified as high. These observations indicate that the model overfits to the dominant label and fails to learn relevant features for the non-dominant labels. Thus, the classification model tends to favor low-pain predictions, as shown by the cluster in the leftmost bar chart in Figure 13.

Next, we look into how the other two biasing techniques affect the classification accuracy for a specific pain level. Applying a band-pass filter refers to muting certain frequency features in the spectrogram, thereby de-emphasizing the features associated with a particular pain level. The central bar graph in Figure 13 demonstrates that applying a band-pass filter enhances the CNN model’s ability to capture features associated with moderate pain, thus increasing the probability of correctly classifying such cases. This approach, which involves amplifying specific frequency components in the spectrogram, effectively emphasizes features that are strongly correlated with particular pain levels. The rightmost group of bars in Figure 13 further illustrates that selectively increasing spectrogram intensity can mitigate the effects of training data imbalance, leading to improved classification accuracy for underrepresented pain levels.

### 4.5. Hardware Prototype Speed

**Figure 14:**
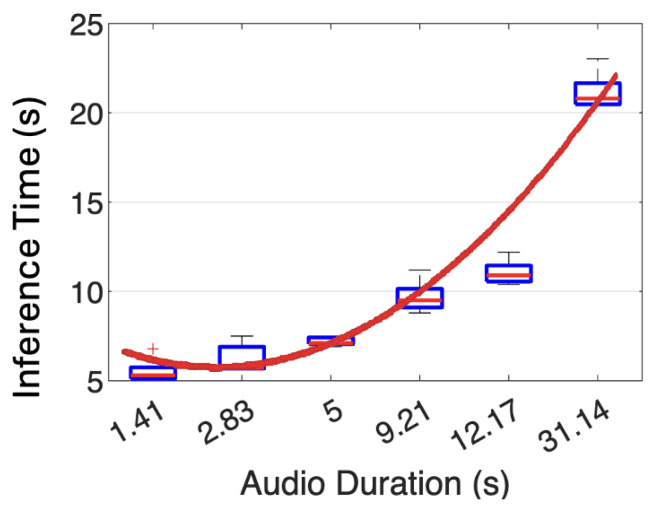
Measured inference times from the hardware prototype for five audio signals with varying durations.

We used five audio signals with varying durations to evaluate the inference times on our hardware prototype. As shown in Figure 14, for an audio duration of 1.41 s, our prototype takes 5.54 s to analyze. When the input audio time increases to 31.14 s, the analysis time rises to 21.19 s. Each audio file was inferred by our trained CNN model five times, yielding an average standard deviation of 0.749 s. We applied a linear regression to obtain a trending line (expressed in Equation (1)) for the average inference time υ against the audio duration time *t*.

(1) $$\upsilon \left(t\right)=0.8704t^{2}-3.3725t+8.758$$

where *x* represents the length of a real-time audio input to the proposed framework.

### 4.6. Impact of Machine Learning Models on Average Accuracy of Pain Level Classification

**Figure 15:**
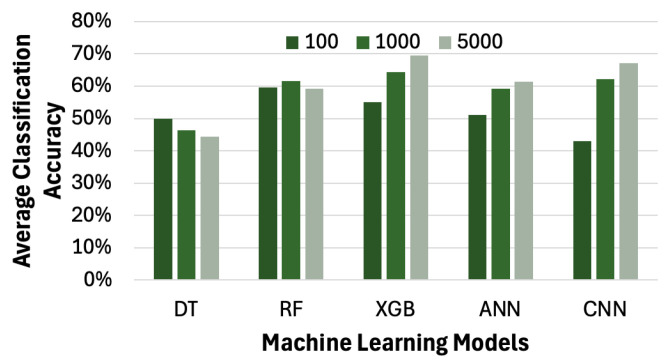
Impact of sample size and machine learning models on average accuracy of pain level classification. DT: decision tree. RF: random forest. XGB: (eXtreme Gradient Boosting). ANN: artificial neural network. CNN: convoluntional neural network.

Since different machine learning models yield varying levels of accuracy in pain level classification, we extend our framework to incorporate several algorithms, including ANN, XGB, RF, and DT. Due to the significantly long training times of certain models, we limit our experiments to datasets with no more than 5000 samples in this subsection. As shown in Figure 15, except for DT, all the other models—RF, XGB, ANN, and CNN—achieve an accuracy of over 60% in classifying the pain level. This experiment confirms that other machine learning models are also applicable in our framework.

## 5. Suggested Real-World Deployment of the Proposed Framework

The experimental results presented in Section 4 suggest that the proposed framework has the potential to support real-world deployment in sensor-assisted intelligent care settings. Given its lightweight design and low computational requirements, the system is intended to operate without continuous professional supervision, making it particularly suitable for home-based and independent living scenarios.

**Figure 16:**
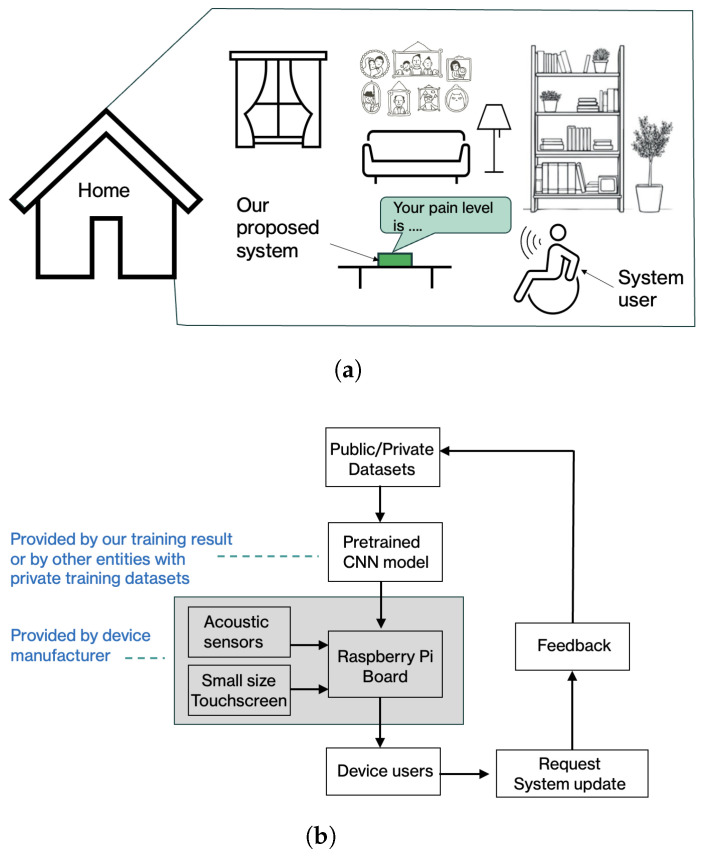
Conceptual real-world deployment scenarios for the proposed voice-based pain classification framework. (**a**) Illustrative example of a home-based usage setting for independent living, where voice signals are captured and processed locally by a stand-alone device, and (**b**) a high-level deployment workflow showing model configuration, optional user feedback, and secure model update mechanisms.

Figure 16a illustrates a conceptual implementation example in a home environment. In this setting, a user interacts with a stand-alone device equipped with an embedded microphone and a pre-configured pain-level classification model, as shown in Figure 7. The voice signals captured by the device are processed locally, and the estimated pain levels are displayed through a simple on-device interface. This design aims to minimize user effort while preserving privacy by avoiding continuous cloud-based data transmission. In an intended usage scenario, the user speaks into the device’s microphone and receives an estimated pain level on the device’s built-in monitor. The device may also include a system update portal that enables the deployment of new CNN models to potentially improve performance over time.

The proposed deployment workflow is summarized in Figure 16b. Device manufacturers or system integrators may deploy a pretrained CNN model provided by this work or adapt the framework using models trained on institution-specific datasets. Model updates can be delivered through a secure update mechanism, allowing system performance to evolve as new data or optimized models become available. User feedback and optional audio samples may be incorporated into future model refinement, subject to appropriate consent and data protection mechanisms.

While the current study focuses on system design and experimental validation using publicly available datasets and a Raspberry Pi-based prototype, future work will explore user-centered evaluations, usability considerations for older adults, and longitudinal assessments in real-world independent living environments. These directions will help further assess the practical effectiveness and acceptance of the proposed framework in sensor-assisted intelligent care applications. Furthermore, pain perception and expression are inherently subjective and may vary significantly between individuals due to personal experiences, education, and cultural norms. In real-world care scenarios, some people may express pain openly through vocal cues, while others may suppress or modify vocal expressions of discomfort. In this work, pain levels are derived from self-labeled pain scores in the adopted datasets, reflecting each subject’s perceived pain experience.

In addition, the proposed framework does not aim to infer an absolute or universal pain threshold. Instead, it focuses on modeling how people express perceived pain through vocal characteristics under labeled conditions. By learning spectral and temporal patterns associated with different self-reported pain levels, the system is intended to provide supportive, complementary information to caregivers and clinicians rather than to replace clinical judgment. Addressing cultural variability and individual differences, such as subject-specific calibration or adaptive learning mechanisms, represents an important direction for future system deployment and personalization. In addition, in real-world deployment, factors such as age, gender, and possible voice pathology can further influence vocal characteristics; addressing these factors through stratified analysis or adaptive modeling also remains an important direction for future work.

Beyond technical feasibility in real-world deployment, it is essential to consider how the proposed model translates into tangible benefits for Independent Living in everyday environments. The proposed technology supports Independent Living by monitoring individual well-being, mitigating communication barriers, and allowing timely, personalized responses without disrupting daily activities. Because the approach is passive and low-cost, it enables more frequent and continuous monitoring, including during nighttime hours, thereby facilitating earlier detection of health deterioration and timely intervention. In regions where access to in-person care is limited by geographic distance or harsh travel conditions, the proposed system offers a practical, scalable alternative that helps individuals remain in their own homes.

In addition, the framework enables neural network models to be trained on user-owned data, potentially better accommodating individual variability and serving users with verbal communication challenges more effectively than generalized clinical assessments. By supporting personalized home-based monitoring and decision support, the proposed technology improves comfort, preserves autonomy, and reduces the reliance on continuous in-person supervision. As a result, it empowers individuals with underlying conditions to manage their daily lives more independently.

In summary, these considerations position the proposed framework not only as a technically feasible system, but also as a practical solution aligned with the core goals of Independent Living.

## 6. Conclusions and Future Work

Advanced healthcare systems that integrate various sensors, edge devices, and machine learning algorithms simultaneously empower assistive living communities. This work contributes to the development of these trending intelligent healthcare systems. As pain can significantly interrupt a person’s normal daily activities, this work focuses on a system for effective pain management. We propose a CNN-based framework for classifying three pain levels. More specifically, we extracted spectral features from verbal and nonverbal voice signals and trained CNN models for both verbal and nonverbal users. Unlike conventional methods that rely on physiological sensors or facial expressions, which often require professional assistance, our system employs user-friendly acoustic sensors to capture audio in real time without the need for precise calibration. Moreover, our hardware prototype can be built for under 100 U.S. dollars and achieves classification speeds of 6–22 s on real-time audio inputs. Our experimental results show that the proposed method achieves over 72% accuracy in classifying three-level pain classification, which is up to 26.74% higher than the existing work with the same classification granularity. Our low-cost, acoustics-sensor-based method achieves a false negative rate in low/high pain classification scenarios comparable with that of the facial expression-based method that relies on a well-positioned camera. Our case studies also examined the impact of sample size and label imbalance on classification accuracy, showing that biased training leads to overfitting and a significant decrease in classification accuracy. The proposed framework enables non-intrusive pain assessment suitable for deployment in low-resource or edge-computing environments.

In real-world deployment scenarios, it is important to recognize that moderate pain represents an inherently transitional and subjective state. As reflected in our experimental results, the classification accuracy for moderate pain is lower than for low and high pain, which can be attributed to overlapping acoustic characteristics across adjacent pain levels and the limited number of moderate pain samples in the training dataset. This limitation highlights a known challenge in multi-class pain modeling, rather than a deficiency of the proposed framework. In many practical independent living applications, reliably identifying clear pain states (e.g., no pain versus severe pain) is often more critical than precisely distinguishing intermediate levels. Future work may explore alternative formulations, such as ordinal classification, data balancing strategies, or transfer learning, to improve the robustness of intermediate pain levels as larger and more diverse datasets become available.

In future work, we also plan to validate the framework in real-world clinical or elder-care settings to evaluate its usability and effectiveness. Although this work focuses on audio-based analysis to preserve simplicity, privacy, and deployability, future research may explore multimodal extensions to improve robustness and contextual awareness. In particular, integrating facial expressions, posture, and motion cues extracted from video signals could provide complementary information for pain assessment, especially when vocal expressions are subtle or suppressed. Sudden changes in posture or movement patterns can serve as additional indicators of discomfort in real-world care settings. In addition, we will consider improving classification accuracy by incorporating hints such as keywords and linguistic features.

## Figures and Tables

**Table 1:** False Negative Rate for Two Methods.

Methods\Comparison	Classification Cue/Label	FNR
Facial expression based	Action Unit 04	0%
	Action Unit 01	9%
	Action Unit 02	18%
	Action Unit 06	27%
	Action Unit 12	36%
Audio-based	Low pain	17.3%
	Moderate pain	46.3%
	High pain	28.7%

**Table 2:** Impact of sample sizes on the probability of predicting the correct pain class.

Samples\True-Predict	Low-Low	Mod-Mod	High-High
100	57.2%	24%	16.7%
500	60.3%	45.9%	4.9%
1000	90%	26.4%	6.6%
5000	73.7%	64.2%	25.3%

**Table 3:** Imbalanced sample distribution for bias study.

Bias Scenario\Samples	Number of Low Pain Samples	Number of Moderate Pain Samples	Number of High Pain Samples
**Bias on low pain**	600	200	200
**Bias on moderate pain**	200	600	200
**Bias on high pain**	200	200	600

## Data Availability

Data is available upon request via email.
